# CYLD Promotes TNF-*α*-Induced Cell Necrosis Mediated by RIP-1 in Human Lung Cancer Cells

**DOI:** 10.1155/2016/1542786

**Published:** 2016-09-25

**Authors:** Xing Lin, Qianshun Chen, Chen Huang, Xunyu Xu

**Affiliations:** Department of Thoracic Surgery, Provincial Clinical College of Fujian Medical University, Fujian Provincial Hospital, Fuzhou, Fujian 350001, China

## Abstract

Lung cancer is one of the most common cancers in the world. Cylindromatosis (CYLD) is a deubiquitination enzyme and contributes to the degradation of ubiquitin chains on RIP1. The aim of the present study is to investigate the levels of CYLD in lung cancer patients and explore the molecular mechanism of CYLD in the lung cancer pathogenesis. The levels of CYLD were detected in human lung cancer tissues and the paired paracarcinoma tissues by real-time PCR and western blotting analysis. The proliferation of human lung cancer cells was determined by MTT assay. Cell apoptosis and necrosis were determined by FACS assay. The results demonstrated that low levels of CYLD were detected in clinical lung carcinoma specimens. Three pairs of siRNA were used to knock down the endogenous CYLD in lung cancer cells. Knockdown of CYLD promoted cell proliferation of lung cancer cells. Otherwise overexpression of CYLD induced TNF-*α*-induced cell death in A549 cells and H460 cells. Moreover, CYLD-overexpressed lung cancer cells were treated with 10 *μ*M of z-VAD-fmk for 12 hours and the result revealed that TNF-*α*-induced cell necrosis was significantly enhanced. Additionally, TNF-*α*-induced cell necrosis in CYLD-overexpressed H460 cells was mediated by receptor-interacting protein 1 (RIP-1) kinase. Our findings suggested that CYLD was a potential target for the therapy of human lung cancers.

## 1. Introduction

Lung cancer, also known as lung carcinoma, is a malignant lung tumor with high morbidity and mortality not only in China but also around the world [1, 2]. There are two main types of lung cancer: non-small cell lung cancer (NSCLC) and small cell carcinoma (SCLC) [3–5]. The common types of NSCLC are including squamous cell carcinoma, large cell carcinoma, and adenocarcinoma [6]. It has been reported that several risk factors contribute to the development of lung cancer, which increase the occurrence of lung cancers. Cigarette smoking is the principal risk factor for development of lung cancer. Especially in China, approximately 67% of males and 4% of females aged over 15 years are smokers, representing more than one-third of all smokers worldwide [7]. Besides, it will lead to the development of lung cancer by exposure to second-hand smoke, radon, arsenic, chromates, nickel, and air pollution, as well as radiation therapy [8, 9].

The triggering of programmed cell death such as cell apoptosis and necrosis among lung cancer cells could have important therapeutic implications. Cylindromatosis (CYLD), a deubiquitinase, is originally identified as a tumor suppressor [10]. Inhibition of CYLD increased resistance to apoptosis by activating NF-kappaB, suggesting a mechanism through which loss of CYLD contributed to oncogenesis in lung cancer cells [11]. CYLD was a key factor to regulate cell survival and cell death, including caspase-8-mediated cell apoptosis and caspase-8 independent cell necrosis. Specifically, CYLD removed lysine 63 and linear ubiquitin chains from RIP1 and promoted necroptosis in TNF receptor signaling, which was involved in the regulation of different cellular processes including inflammation, fibrosis, and cancer. Koga et al. had reported that CYLD interacted with and deubiquitinates TAK1 by negatively regulating the activation of the downstream MKK3/6-p38alpha/beta pathway to resist the infection of gram-positive bacterium* Streptococcus pneumonia* [12]. CYLD also inhibited inflammation and proliferation in vascular cells and represented a novel target for the treatment or prevention of atherosclerosis [13]. Wang et al. have found that the BRG1- and hBRM-associated factor BAF57 induced apoptosis by stimulating expression of the cylindromatosis tumor suppressor gene and increased expression of CYLD in BT549 cells induced apoptosis [14].

Recently, it has been found that familial CYLD mapping on 16q12-q13 was an autosomal dominant genetic predisposition to multiple tumors of the skin appendages [10, 15]. Hellerbrand and Massoumi have found that mutation or disruption of the activity of CYLD in animals aggravated acute as well as chronic liver injury and promoted development and progression of hepatocellular cancer [16]. Deletion of exon 9 of CYLD would cause a carboxyl-terminal truncation of CYLD and inactivation of its deubiquitinating activity, which has been associated with the maturation of lung [17]. Downregulation of CYLD induced tumor cell proliferation and consequently contributed to the aggressive growth of hepatocellular carcinoma [18]. Hayashi et al. have found that CYLD downregulation promoted breast cancer metastasis via NF-kappaB activation, including RANKL signaling [19]. However, the role of CYLD in lung cancer was not clearly clarified. In the present study, we explored the role of CYLD in human lung cancer specimens and the molecular mechanism of CYLD was investigated in the progression and development of human lung cancers.

## 2. Material and Method

### 2.1. Patients

The study was conducted over a period of 24 months from May 2012 to May 2014. A total of 19 patients (11 men and 8 women) were included in the study with the median age of 76.53 years (range 49–76 years). All the patients were given a precise pathology diagnosis of non-small lung cancers. The samples were obtained from surgery and the patients were not given radiotherapy or chemotherapy before. The fresh tissues were quickly frozen in liquid N_2_ and kept in refrigerator at −80°C, which was used for detecting CYLD expression by real-time PCR and western blotting analysis. The lung carcinoma specimens and the paired paracarcinoma tissues were obtained from the consenting patients in Fujian Provincial Hospital. The patients were well informed and signed the relevant contracts prior to the experiment and the experiment was approved by the Ethics Committee of Fujian Provincial Hospital.

### 2.2. Cell Lines and Agents

Human lung adenocarcinoma cell line A549 (Cat. number TcHu150) and large cell lung cancer cell line H460 (Cat. number TcHu205) were purchased from Cell Resource Center of Shanghai Institutes for Biological Sciences, Chinese Academy of Sciences. The lung cancer cells were cultured in DMEM medium with 10% fetal bovine serum, 1% penicillin, and 1% streptomycin. Three pairs of CYLD siRNA and negative control siRNA were purchased from Abm Corporation (Richmond, BC, Canada) and the catalogue number was i505598. The RIP-1 siRNAs were designed and synthetized by Jima Corporation, Shanghai, China. The sequences of the siRNAs specific to RIP-1 used were as follows:

 Human RIP1: 5′-UGCUCUUCAUUAUUCAGUUUGCUCCAC-3′;
 human RIP1: 5′-UGCAGUCUCUUCAACUUGAAdTdT-3′.

MTT agent was purchased from Sigma Inc. (Sigma, Saint Louis, MO). The pcDNA3(+)/CYLD-flag plasmid and negative control plasmid pcDNA3.1(+) were kept in our laboratory. Caspase Inhibitor carbobenzoxy-valyl-alanyl-aspartyl-[O-methyl]-fluoromethylketone (z-VAD-FMK) was obtained from Promega Company with catalogue number of G7231. The recombinant human TNF-*α* (Cat. number 10602-HNAE-10) consisted of 158 amino acids with the molecular mass of 17.4 kDa and was obtained from Sino Biological Incorporation (Beijing, China). Necrostatin-1 (Cat. number N9037-10MG) was purchased from Sigma Corporation.

### 2.3. Real-Time PCR Assay for CYLD Detection

The specimens from lung cancer tissues and paired paratumor tissues were prepared as described above. The total RNAs in each sample were extracted with an RNApure kit (Bioteke, Beijing, China). All the RNA samples were retrotranscribed with MLV-reverse transcriptase (Invitrogen Inc., Carlsbad, USA). Quantitative real-time PCR was performed on an Applied Biosystems 7500 Real-Time PCR System (ABI, Foster City, USA). The sequences of the primers were used for real-time PCR amplifications as follows: for CYLD: 5′-TGCCTTCCAACTCTCGTCTTG-3′ and 5′-AATCCGCTCTTCCCAGTAGG-3′; for beta-actin: 5′-ACTCGTCATACTCCTGCT-3′ and 5′-GAAACTACCTTCAACTCC-3′. Each cDNA sample was made in triplicate. The thermal cycling conditions were 40 cycles of 12 s at 95°C, 12 s at 65°C, and 5 s at 72°C using SYBR Green.

### 2.4. Transient Transfection of Human Lung Cell Lines with CYLD-Flag Plasmid and Control Plasmid

Cell clones expressing CYLD were established by transient transfection using lipofectamine plus (Invitrogen) in human lung cancer cell lines A549 and H460, respectively. Twenty-four hours after transfection, cells were collected and analyzed by western blotting analysis. The positive clones for overexpression of CYLD are screened. Briefly, an increasing concentration of G418 was designed to treat CYLD-flag-transfected cells or pcDNA3.1 plasmid-transfected A549 and H460 cells. The final concentration of G418 was 0, 200, 400, 600, 800, and 1000 *μ*g/mL, respectively. The transfected cells were treated and killed with the indicated concentration of G418 in a week, which was identified as the appropriate concentration for screening the stable cell line. Next, the transfected cells were plated into the 6-well plate as less as possible. The cells were treated with the appropriate screening concentration of G418 for 24 hours till the death of negative control cells. Here, the untreated lung cancer cells A549 or H460 were used as negative control. Finally, the survival clones were proliferated and the CYLD expression was detected by western blotting analysis. The stable transfected cells were kept in our laboratory.

### 2.5. Flow Cytometric Analysis

The stable cell line of CYLD-overexpressing A549 cells was treated with 10 ng/mL of TNF-*α* alone, or 20 ng/mL of TNF-*α* combinated with 10 *μ*M of a pancaspase inhibitor, z-VAD-fmk, for 12 hours, and the cell apoptosis rate and necrosis rate were determined by Annexin v-FITC/PI dual staining analysis following the kit protocols (Santa Cruz, USA). Briefly, the cells were washed in PBS buffer and resuspended in binding buffer (HEPES–NaOH 10 mM pH 7.4, 25 mM CaCl_2_, and 144 mM NaCl). Then, the staining dye of Annexin V (0.1 *μ*g/*μ*L) and PI (0.05 *μ*g/*μ*L) was added and incubated in dark for 30 min on ice. Finally, more than 3 × 10^5^ cells were prepared and subjected to FACS analysis.

In the other treatment, the stable cell line H460 cells with overexpression of CYLD-flag or negative control plasmid were treated with TNF-*α* at the increasing concentration of 0 ng/mL, 1 ng/mL, 20 ng/mL, and 400 ng/mL for 20 hours. The cells were washed in PBS buffer and resuspended in binding buffer (HEPES–NaOH 10 mM pH 7.4, 25 mM CaCl_2_, and 144 mM NaCl). The PI (0.05 *μ*g/*μ*L) was added and incubated in dark for 5 min on ice and the necrosis rate was determined by FACS assay with PI staining.

### 2.6. MTT Assay

The cell survival rate and cell proliferation were determined by MTT assay as described. The A549 cells and H460 cells were plated into 48-well plates and the cells were transfected with CYLD specific siRNA or negative control siRNA for 24 h, 48 h, and 72 h, respectively. Four hours before test, 5 mg/mL of MTT agent was added into the medium. Before test, the purple crystals were dissolved with 100 *μ*L of DMSO for 10 min. The data was tested at a test wavelength of 490 nm.

### 2.7. Western Blot

The samples were prepared by RIPA-buffer (Roche) and whole protein concentration was determined by the BCA protein assay reagent (Pierce, USA). The proteins in cell lysates were separated by 10% SDS-PAGE and transferred onto PVDF membranes (Bio-Rad, Richmond, USA) at constant electric current of 400 mA for 1 h. The membranes were blocked in 5% BSA/PBS for 30 min, and the membrane was incubated with related primary antibodies at 4°C overnight. The antibodies involved in the present study were as follows: rabbit polyclonal CYLD antibody (Catalog number: 11110-1-AP) was obtained from Proteintech Corporation (Wuhan, Hubei, China). anti-RIP-1 antibody (Cat. number ab2035) was obtained from Abcam Corporation, which was corresponding to a region between amino acids 420 and 433 of Human RIPK1 and was a rabbit polyclone antibody. Beta-actin antibody (Cat. number AM1829B) was a purified mouse monoclonal antibody supplied in PBS with 0.09% (W/V) sodium azide and obtained from Abgent Corporation. Next, the membranes were washed three times in PBS buffer (5 min each time). The horseradish peroxidase-conjugated goat anti-rabbit secondary antibody was cultured for 30 min at room temperature, which was obtained from Santa Cruz Biotechnology, Inc. After development, the bands were analyzed in each lane.

### 2.8. Statistical Analysis

The data were analyzed by SPSS software. Student's *t*-test was used to evaluate statistical significance. Data was shown as mean value ± SD. Value of *p* < 0.01 was considered to be significant.

## 3. Result

### 3.1. Lower Levels of CYLD Are Detected in Clinical Lung Carcinoma Specimens

**Figure 1:**
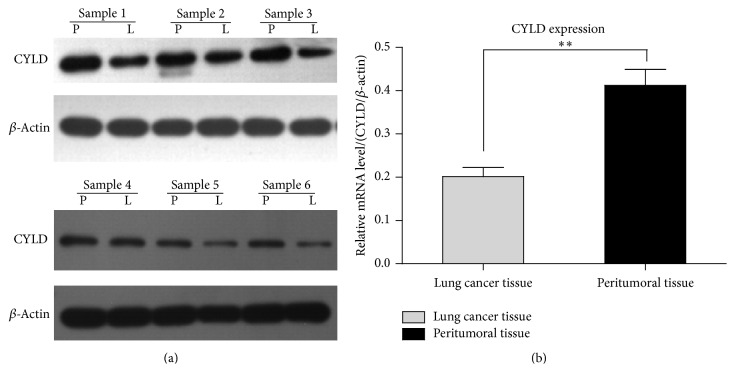
Lower levels of CYLD are detected in clinical lung carcinoma specimens. The surgical specimens including lung cancer tissues and the paired peritumoral tissues from the lung cancer patients were collected. (a) The expression levels of CYLD were detected by western blotting analysis. The data shown here was representative of six independent specimens. The housekeeping gene, *β*-actin, was used as an internal reference in the experiment. P, paracarcinoma tissue; L, lung adenocarcinoma tissue. (b) The levels of CYLD were detected by real-time PCR assay. Ratios of CYLD/*β*-actin were shown representing the relative mRNA levels in lung cancer patients. The data was shown as mean values ± SD. ^*∗∗*^ *p* < 0.01 compared with peritumoral tissue.

In order to investigate the clinical significance of* CYLD* expression,* CYLD* gene expressions were analyzed in 19 lung carcinoma specimens and the paired paracarcinoma tissues by real-time PCR and western blotting. In the present study, 6 representative cases were shown in Figure 1. In the 6 surgical specimens from the patients diagnosed with lung adenocarcinoma, CYLD expressions were detected in both lung adenocarcinoma tissues and peritumoral tissues. Compared to paracarcinoma tissues, the relative CYLD expressions in mRNA levels and protein levels were significantly decreased in lung carcinoma compared to that in the paired paracarcinoma tissues (^*∗∗*^ *p* < 0.01). Here, *β*-actin was used as the internal reference. The data was normalized to *β*-actin and expressed as mean value ± SD. All of the data demonstrated that CYLD might work as a tumor suppressor in the progression and development of lung cancer.

### 3.2. Knockdown of CYLD by siRNA Promotes Cell Proliferation of A549 and H460 Cells

**Figure 2:**
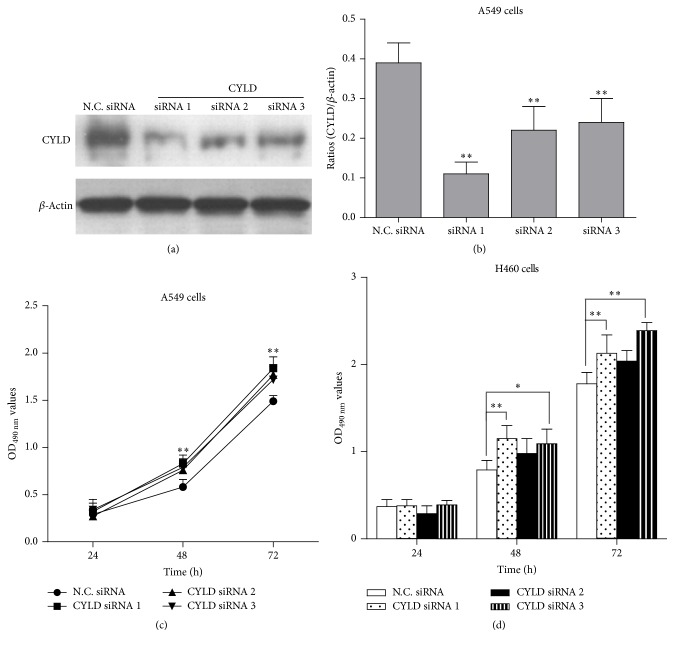
Knockdown of CYLD by siRNA promotes the growth of A549 and H460 cells. (a) The A549 cells were plated into 24-well plate. Eight hours later, three pairs of CYLD-siRNA were transfected into A549 cells. The transfected cells were cultured for 48 hours; the expression of CYLD was detected by western blotting analysis. (b) *β*-Actin was used as an internal reference and N.C. siRNA was used as the negative control. The expression level of CYLD was normalized to the expression of *β*-actin. ^*∗∗*^ *p* < 0.01, compared with the N.C. siRNA transfected group. (c) Cell viability was determined by MTT assay. The transfected A549 cells were cultured for 24 hours, 48 hours, and 72 hours, respectively, and the OD_490 nm_ values were detected by MTT assay. ^*∗∗*^ *p* < 0.01, compared with N.C. siRNA group. (d) H460 cells were transfected with three pairs of siRNA specific to CYLD and cultured for 24 h, 48 h, and 72 h, respectively. The cell viability was determined by MTT assay. ^*∗*^ *p* < 0.05, ^*∗∗*^ *p* < 0.01, compared with N.C. siRNA group.

In order to further identify the function of CYLD in lung carcinoma cells, transient CYLD-knockdown A549 cells were generated using three pairs of siRNA specific to CYLD. The control siRNA- (N.C. siRNA-) transfected cells were used as negative controls. As shown in Figure 2(a), CYLD-siRNA efficiently downregulated the levels of CYLD in lung cancer A549 cells by western blotting. Moreover, the interference effects of the siRNAs specific to CYLD were 71.8%, 43.6%, and 38.5%, respectively (Figure 2(b)). Next, three pairs of CYLD-siRNAs were used to transfect the lung cancer cell lines and cell proliferation was determined by MTT assay. As shown in Figures 2(c) and 2(d), the cell viability was significantly increased in CYLD-knockdown A549 cells and H460 cells compared to that in N.C. siRNA-transfected cells after transfection for 48 hours and 72 hours (^*∗*^ *p* < 0.01, ^*∗∗*^ *p* < 0.05, compared with N.C. siRNA-transfected cells) suggesting knockdown CYLD in lung cancer cells obviously promoted cell proliferation of A549 and H460 cells.

### 3.3. Transfection of pcDNA3.1(+)-CYLD Plasmid Increases CYLD Expression and Promotes Cell Death in Lung Cancer Cell Lines

**Figure 3:**
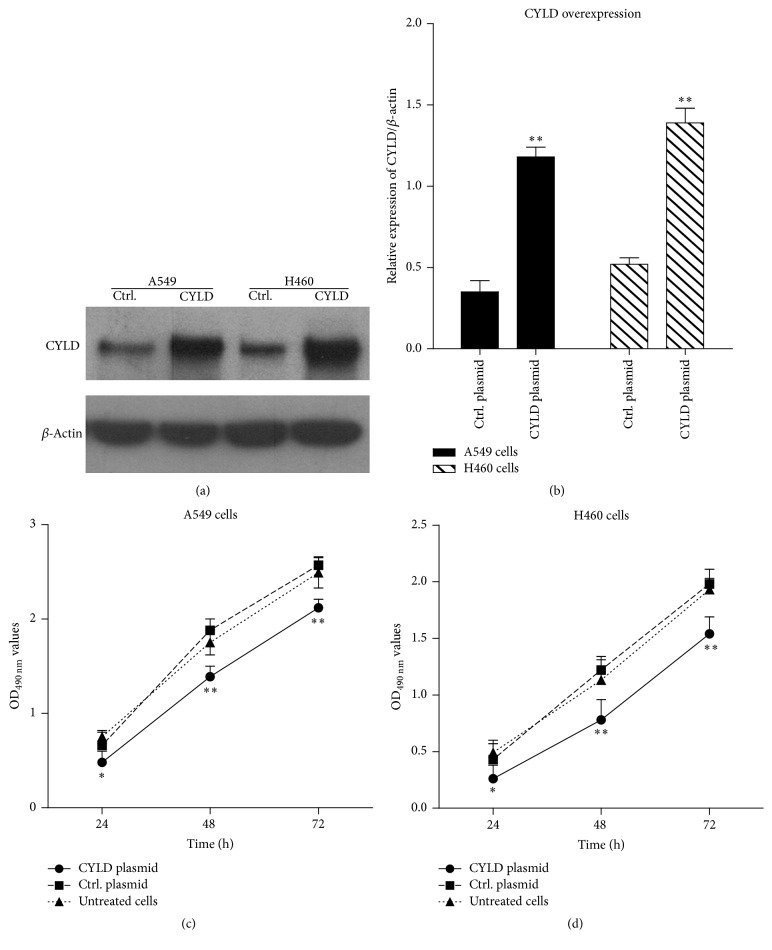
Transfection of pcDNA3.1(+)-CYLD plasmid increases CYLD expression in lung cancer cell lines. (a) The lung cancer cells A549 and H460 were transfected with the recombinated plasmid pcDNA3(+)/CYLD-flag and control plasmid pcDNA3.1(+) for 24 hours. The levels of CYLD were detected by in lung cancer cell lines western blotting analysis. (b) The images were captured by image J software and gray values were analyzed and shown in histogram. ^*∗∗*^ *p* < 0.01, compared with control plasmid. (c) A549 cells were transfected with CYLD-flag plasmid and cultured for 24 h, 48 h, and 72 h, respectively. The cell viability was determined by MTT assay. ^*∗*^ *p* < 0.05, ^*∗∗*^ *p* < 0.01, compared with the control plasmid transfected cells. (d) H460 cells were transfected with CYLD-flag plasmid for 24 h, 48 h, and 72 h. The cell viability was determined by MTT assay. ^*∗*^ *p* < 0.05, ^*∗∗*^ *p* < 0.01, compared with the control plasmid transfected cells.

Further, the lung cancer cell lines A549 and H460 were transfected with pcDNA3(+)/CYLD-flag plasmid and control plasmid for 24 hours. The levels of CYLD were obviously increased in CYLD-flag plasmid transfected A549 cells and H460 cells (Figure 3(a)) by western blotting analysis. The relative expression levels of CYLD following CYLD overexpression in A549 cells and H460 cells were significantly higher (3.37 ± 0.16 and 2.67 ± 0.17 times), compared with that in pcDNA3.1 plasmid transfected A549 and H460 cells. Accordingly, the cell proliferation was also determined by MTT assay. As shown in Figures 3(c) and 3(d), cell proliferation of A549 cells or H460 cells was significantly decreased compared to that of untreated cells (^*∗*^ *p* < 0.05, ^*∗∗*^ *p* < 0.01). All of the results demonstrated that overexpression of CYLD promoted cell death and CYLD played an antitumor activity in the human lung cancer cells.

### 3.4. Overexpression of CYLD-Flag Induces Cell Necrosis of Lung Cancer Cells

**Figure 4:**
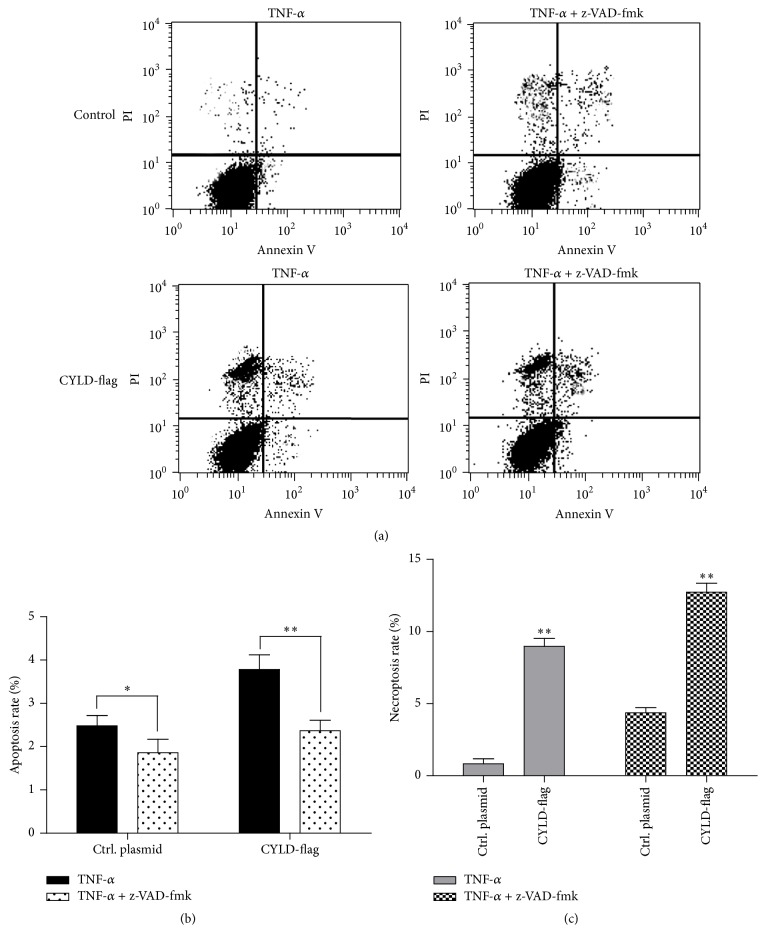
Overexpression of CYLD-flag induces cell necrosis of H460 cells. (a) Induction of apoptosis and necrosis was determined by Annexin V-PI dural staining. The A549 stable cell lines with CYLD expression and the control cell line were plated into 12-well plate and treated with 10 ng/mL of TNF-*α* alone, 20 ng/mL of TNF-*α* combinated with 10 *μ*M of a pancaspase inhibitor, z-VAD-fmk for 12 hours. The 2 × 10^6^ cells were collected and washed by PBS buffer. Annexin v-FITC/PI dual staining analysis was determined as the Material and Method. (b) The apoptosis rate of lung cells transfected with CYLD-flag or control plasmid was shown in histogram. ^*∗*^ *p* < 0.01, compared with control plasmid. ^*∗∗*^ *p* < 0.01, compared with N.C. siRNA group. (c) The necrosis rate was analyzed and shown in histogram. Values were mean ± SD. ^*∗∗*^ *p* < 0.01, compared with control plasmid.

Next, the mechanism of antitumor activity for CYLD was further to be explored. Firstly, we asked whether overexpression of CYLD-flag could induce cell apoptosis, which probably contributed to CYLD-induced cell death. As shown in Figure 4(a), A549 cells were treated with 10 ng/mL of TNF-*α* alone, or 20 ng/mL of TNF-*α* combinated with 10 *μ*M of a pancaspase inhibitor, z-VAD-fmk, for 12 hours. The cell apoptosis rate was determined by Annexin v-FITC/PI dual staining analysis. The results demonstrated that after treatment with 10 ng/mL of TNF-*α* for 12 hours cell apoptosis rate was increased in TNF-*α* treated group compared with that in TNF-*α* plus z-VAD-fmk treated group (^*∗*^ *p* < 0.05, ^*∗∗*^ *p* < 0.01). Moreover, overexpression of CYLD increased TNF-*α* induced cell apoptosis in A549 cells. Surprisingly and interestingly, after treatment with TNF-*α* or TNF-*α* plus z-VAD-fmk, the PI-positive rate was significantly in CYLD-transfected cells (^*∗∗*^ *p* < 0.01, compared with Ctrl. Plasmid transfected cells), suggesting that overexpression of CYLD was probably contributed to the cell necrosis in lung cancer cells.

### 3.5. Overexpression of CYLD Enhances TNF-*α*-Induced Cell Necrosis of Lung Cancer Cells

**Figure 5:**
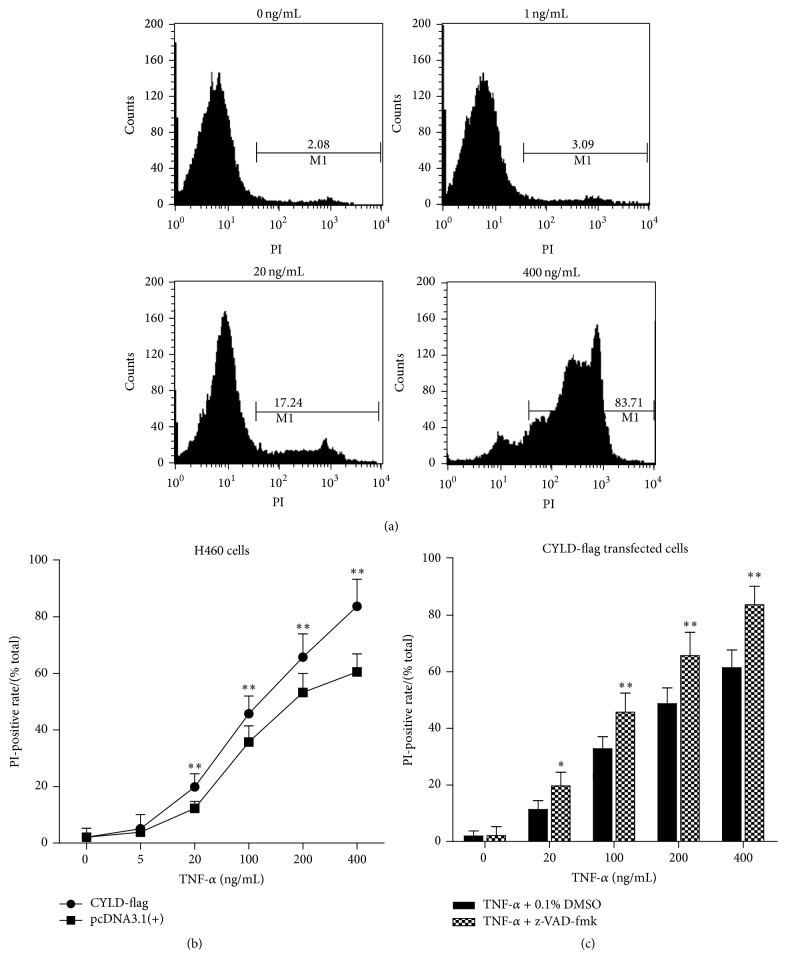
Overexpression of CYLD enhances TNF-*α*-induced cell necrosis of lung cancer cells. (a) The necrosis was determined by FACS assay with PI staining. The CYLD-flag plasmid was used to transfect the lung cancer cell line H460 cells. The stable transfected cell line was screened as described in Material and Method. Increasing concentration of TNF-*α*, including 0 ng/mL, 1 ng/mL, 20 ng/mL, and 400 ng/mL, respectively, was used to treat the CYLD-flag plasmid transfected H460 cells. (b) The PI-positive rates of CYLD-flag transfected H460 cells were shown in histogram. ^*∗∗*^ *p* < 0.01, compared with pcDNA3.1(+) transfected H460 cells. (c) The CYLD-flag transfected cells were pretreated with a pancaspase inhibitor, z-VAD-fmk at the concentration of 10 *μ*M for 6 hours, and different concentrations of TNF-*α* were used to treat H460 to induce cell necrosis. The PI-positive rates of H460 cells were shown in histogram. ^*∗*^ *p* < 0.05, ^*∗∗*^ *p* < 0.01, compared with the group without z-VAD-fmk.

TAB-TAK1-NF-*κ*B complex induces cell survival in the TNF-*α*/TNF-R1 signaling pathway; however, CYLD is a negative regulator of NF-*κ*B signaling pathway which contributes to cell apoptosis and necrosis. Here, the lung cancer cell line H460 cells were transfected with CYLD-flag plasmid and treated with increasing concentrations (0 ng/mL, 1 ng/mL, 20 ng/mL, and 400 ng/mL) of TNF-*α* for 24 hours. As shown in Figure 5(a), cell necrosis was determined by FACS assay with PI staining and the result demonstrated that necrosis rate was significantly increased as the increasing concentrations of TNF-*α*. Moreover, the necrosis rate was significantly higher in CYLD-overexpressed H460 cells than that of pcDNA3.1 plasmid transfected H460 cells (^*∗∗*^ *p* < 0.01) (Figure 5(b)). Additionally, as shown in Figure 5(c), the necrosis rate was significantly increased in TNF-*α* and z-VAD-fmk treated cells compared to that of the cells treated with TNF-*α* alone (^*∗*^ *p* < 0.05, ^*∗∗*^ *p* < 0.01). All of the data revealed that overexpression of CYLD promoted cell necrosis in lung cancer cells.

### 3.6. TNF-*α*-Induced Cell Necrosis Is Mediated by Receptor-Interacting Protein 1 (RIP-1) Kinase

**Figure 6:**
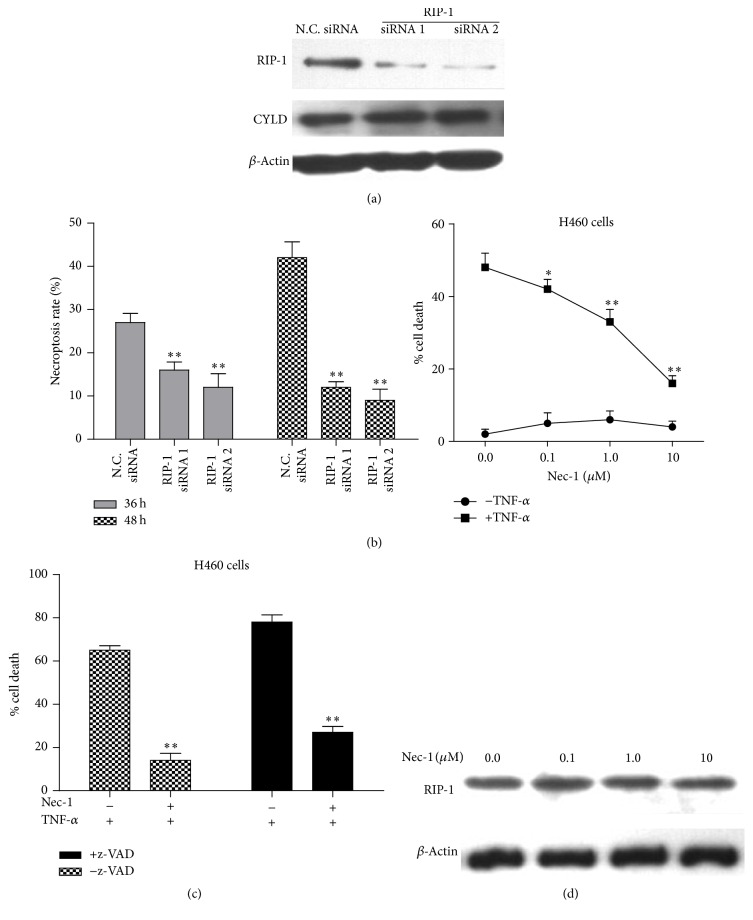
TNF-*α*-induced cell necrosis in CYLD-overexpressed H460 cells is mediated by receptor-interacting protein 1 (RIP-1) kinase. (a) The stable cell line of CYLD-flag transfected H460 cells was plated into 6-well plate. The cells were cultured for 6 hours and transfected with two pairs of siRNA specific to RIP-1 for 48 hours. The levels of endogenous RIP-1 and CYLD were detected by western blotting analysis. *β*-Actin was used as internal reference gene in the present experiment. (b) The CYLD-overexpressed H460 cells were transfected with siRNA specific to RIP1 for 36 h and 48 h. The necrosis rate (PI-positive rate) was determined by FACS analysis. ^*∗∗*^ *p* < 0.01, compared with the cells transfected with N.C. siRNA. (c) The H460 cells were plated into 12-well plate and treated with 100 ng/mL of TNF-*α* in combination with or without 10 *μ*M of z-VAD-fmk for 24 hours. Then, the cells were collected and PI-positive cell rate was determined as described in Material and Method. ^*∗∗*^ *p* < 0.01, compared with the group without z-VAD. (d) The CYLD-overexpressed H460 cells were plated into 6-well plate and treated with 100 ng/mL of TNF-*α* in combination with increasing concentrations of Nec-1 for 24 hours. The concentrations of Nec-1 were 0 *μ*M, 0.1 *μ*M, 1.0 *μ*M, and 10 *μ*M, respectively. The expression levels of RIP-1 were determined by western blotting analysis.

RIP-1 is recognized as a key upstream regulator that controls inflammatory signaling as well as the activation of cell apoptosis and necrosis, which is tightly controlled by ubiquitylation and deubiquitylation. In order to determine whether TNF-*α*-induced cell necrosis was dependent on RIP-1 in CYLD-overexpressed H460 cells, two pairs of siRNA specific to RIP-1 were designed to interfere with endogenous RIP-1 in CYLD-flag transfected H460 cells. As shown in Figures 6(a) and 6(b), RIP-1 expression was obviously reduced and the necrosis rate was significantly decreased in RIP-1 siRNA transfected cells (^*∗∗*^ *p* < 0.01, compared with N.C. siRNA-transfected H460 cells).

Moreover, CYLD-overexpressed H460 cells were treated with 100 ng/mL of TNF-*α* in combination with or without 10 *μ*M of z-VAD-fmk for 24 hours. Necrostatin-1 (Nec-1) inhibited cell necrosis by allosterical inhibition on the kinase activity of RIP-1. In the other treatment, 10 *μ*M of Nec-1 was simultaneously added into the cells, as shown in Figure 6(c), and the cell necrosis rate was significantly decreased compared with that of the group without z-VAD-fmk (^*∗∗*^ *p* < 0.01). Furthermore, the CYLD-overexpressed H460 cells were treated with 100 ng/mL of TNF-*α* in combination with different concentrations of Nec-1 for 24 hours, including 0 *μ*M, 0.1 *μ*M, 1.0 *μ*M, and 10 *μ*M, respectively (Figure 6(d)). As the increasing concentrations of Nec-1, the necrosis rate was significantly decreased compared with that of the cells without Nec-1 (^*∗*^ *p* < 0.05, ^*∗∗*^ *p* < 0.01). Interestingly, the expression levels of RIP-1 were not obviously changed as the increasing concentration of Nec-1, suggesting that the Nec-1 treatment did not affect the endogenous expression of RIP-1 protein (Figure 6(d)).

## 4. Discussion

Lung cancer is one of the most common cancers in the world. The currently available therapies for advanced or metastasized lung cancer have limited outcomes and side effects on overall survival [20, 21]. Therefore, it is important to clarify the clear and exact molecular mechanisms on the underlying onset and progression and involving signaling pathway of lung carcinoma. Additionally, the new potential therapeutic strategy for lung cancer therapy is to be explored. Generally, the pathogenesis of lung cancer is involved in a complex and multistep process, especially the activation of pro-oncogenes and the inactivation of tumor suppressor genes. It has reported that CYLD was a tumor suppressor that regulated cell apoptosis and necroptosis by acting as a deubiquitinating enzyme [22]. It also interacted with other signaling pathway, such as NF-kappaB pathway [11, 23, 24], JNK/AP1 signaling pathway [25], toll-like receptor 2 signaling [26], and notch signaling [27]. Previous studies detected that downregulation or loss of the CYLD gene in human breast cancers [19] and chronic lymphocytic leukemia [28] and mutations of CYLD caused two clinically distinct cancer syndromes [29]. Deng et al. have found that, in human lung cancer cells, overexpressing CYLD augmented antitumor activity of TRAIL by inhibiting the NF-kappaB survival signaling [30]. The aim of the present study is to explore whether and how CYLD regulates cell necrosis in lung cancer cells and further clarifies the molecular mechanism of CYLD in the development of lung cancers.

Firstly, we detected the CYLD expression by real-time PCR and western blotting analysis in 39 lung carcinoma specimens and the paired paracarcinoma tissues. The result obviously revealed that downregulation of CYLD was observed in lung cancer specimens, and we thought that CYLD might work as a tumor suppressor in cancer development and progression [11, 31]. This was consistent with the results by Zhong et al. [32], and they demonstrated that four genes, including cylindromatosis, CD9, activating transcription factor 3, and oxytocin receptor, were dominantly regulated by histone deacetylation and were also frequently downregulated in lung tumors. Next, three pairs of CYLD specific siRNAs were designed to knock down the endogenous CYLD in human lung cancer cell lines A549 and H460. MTT assay results demonstrated that interfering with the expression of CYLD significantly increased the proliferation of A549 and H460 cells (^*∗∗*^ *p* < 0.01, compared with the N.C. siRNA-transfected cells). Conversely, we constructed an expression vector pcDNA3.1(+)-CYLD plasmid and lung cancer cells were transfected with pcDNA3.1(+)-CYLD to increase the levels of CYLD in lung cancer cells. As we expected, the MTT assay results showed that overexpression of CYLD obviously decreased the proliferation of A549 cells or H460 cells compared with that of untreated cells (^*∗*^ *p* < 0.05, ^*∗∗*^ *p* < 0.01). This was consistent with the result that CYLD upregulation contributed to the degradation of ubiquitin chains on RIP1 and subsequent caspase-8 activation and apoptosis [33].

We have detected that overexpression of CYLD contributed to cell death of the lung cancer cells. We further wanted to identify whether the cell death was due to cell apoptosis or necrosis. As we have known, the complex IIa, including RIP1, TRADD, FADD (FAS-associated death domain), RIP3, pro-caspase-8, and FLICE inhibitory proteins (FLIP), was important for cell apoptosis, while the formation of complex IIb, including caspase-8, FADD, RIP1, RIP3, and mixed lineage kinase domain-like (MLKL) protein, led to necroptosis [34]. In the present study, treatment with caspase inhibitor zVAD-fmk obviously increased TNF-*α*-induced cell necrosis. We also detected that overexpression of CYLD increased TNF-*α*-induced programmed cell apoptosis and necrosis. Polyubiquitinated RIP1 was a known substrate of CYLD, a deubiquitinase involving the process of cell apoptosis and necrosis signaling. Importantly, pretreatment with z-VAD-fmk significantly increased the cell necrosis which was dependent on RIP1 kinase. Additionally, Nec-1, the RIP-1 inhibitor, also suppressed the apoptosis rate in TNF-*α*-induced cell necrosis, but it did not affect the expression of RIP-1 in CYLD-overexpressed lung cancer cells. Thus, the reconstitution of CYLD could be an effective and promising method for human lung cancer therapy.
